# The Effects of Online Yoga Practice on Cancer Patients: A Systematic Review

**DOI:** 10.3390/healthcare13030225

**Published:** 2025-01-23

**Authors:** Francesca Gatti, Gaia Perego, Francesca Milano, Gloria Calleri, Bianca Giurioli, Valentina Elisabetta Di Mattei

**Affiliations:** 1 School of Psychology, Vita-Salute San Raffaele University, 20132 Milan, Italy; francescagatti.mail@gmail.com (F.G.); perego.gaia@hsr.it (G.P.); dimattei.valentina@hsr.it (V.E.D.M.); 2 Clinical and Health Psychology Unit, IRCCS San Raffaele Scientific Institute, 20132 Milan, Italy; callerigloria@gmail.com (G.C.); bianca.giurioli@hotmail.com (B.G.); 3 Department of Psychology, University of Milano-Bicocca, 20132 Milan, Italy

**Keywords:** oncology, online yoga, telehealth, cancer side effects, non-pharmacological interventions, complementary therapies, systematic review

## Abstract

**Background**: Cancer remains a leading cause of death, with 9.7 million deaths in 2022. Despite advancements in diagnosis and treatment, many cancer patients experience side effects that significantly impact their quality of life, including chronic pain, anxiety, depression, sleep disturbances, and cancer-related fatigue. Non-pharmacological interventions, such as yoga, have gained attention for their potential to reduce stress and improve overall well-being. However, barriers such as fatigue, pain, and transportation issues limit access to in-person yoga, leading to the growing adoption of online yoga as a viable alternative. **Objective**: This systematic review synthesizes research on the effectiveness of online yoga for cancer patients. A comprehensive search was conducted across Medline, PsycINFO, and Scopus databases on 24 October 2024. The methodological quality of the studies was assessed using the CASP Checklist. Of 6266 articles initially identified, 14 studies met the inclusion criteria, comprising qualitative (n = 4) and quantitative (n = 10) studies. **Results**: The results suggest that online yoga can improve stress and sleep quality, with moderate effects on anxiety, depression, and fatigue. However, variability in study designs and methodological limitations complicate the evaluation of its overall effectiveness. **Conclusions**: Online yoga offers a practical, accessible option for cancer patients unable to attend in-person sessions, showing the potential to enhance mental and physical health outcomes. Nevertheless, the variability in study methodologies highlights the need for more standardized research to establish its role as a supportive intervention in oncology care.

## 1. Introduction

Cancer is one of the leading causes of death globally, with 9.7 million deaths in 2022 [1]. However, according to the World Health Organization (WHO), the global survival rate for all types of cancer is around 50% worldwide, with considerable variation between high-income and low-income regions. Despite these advancements in diagnosis and treatment, many patients endure debilitating side effects that significantly impact their quality of life (QoL) [2,3], defined as an individual’s perception of their position in life, influenced by goals, expectations, and cultural and social factors [4]. Indeed, the challenges of cancer extend beyond physical symptoms, profoundly affecting patients’ psychological, social, and emotional well-being [5,6].

Many cancer patients experience chronic pain, anxiety, depression, and sleep disturbances, which affect treatment adherence and survival outcomes [7,8]. Additionally, they often face cancer-related fatigue (CRF), a multifaceted condition that further diminishes physical, emotional, and cognitive well-being [9].

Non-pharmacological therapies, such as psychological interventions, have proven effective in reducing distress and improving QoL [10]. Building on this evidence, the adoption of a multidisciplinary approach that integrates complementary therapies delivered by diverse healthcare professionals further amplifies these benefits, leading to significant improvements in QoL [11]. Complementary and alternative medicine (CAM), including treatments like meditation, mindfulness, and relaxation techniques, has gained popularity [12,13] and is increasingly integrated into healthcare systems worldwide [14].

Among these, yoga stands out as a beneficial practice, promoting stress reduction, improved immunity, and overall well-being [15]. Through physical postures and breathing techniques, yoga stimulates the parasympathetic nervous system, reducing stress hormones and positively influencing psychological and cognitive health [16].

Yoga for cancer patients has been shown to alleviate symptoms such as depression, anxiety, and fatigue [17], with systematic reviews suggesting physical, psychological, and cognitive improvements [18,19]. However, not all individuals can practice yoga in person due to barriers such as fatigue, pain, transportation issues, and scheduling conflicts; in such cases, online yoga offers a potentially beneficial alternative [20]. Indeed, online yoga provides individuals the flexibility to practice at their convenience, eliminating the need for travel and associated costs. This accessibility has been shown to reduce missed sessions and increase satisfaction [21]. Yoga can be practiced at home with minimal expenses and equipment, making it a cost-effective and adaptable option that caters to individual needs and abilities [22]. Particularly during the COVID-19 pandemic, online yoga has emerged as a valuable alternative, allowing individuals, especially those unable to attend in-person sessions, to continue their practice from home [22,23].

To better understand the potential benefits of online yoga in oncology, comparing them with evidence on traditional in-person yoga is helpful. For instance, a systematic review analyzed 34 randomized controlled trials and found that traditional yoga improves physical function, mental health, and overall quality of life in the short term [24]. These findings align with the previous literature [18,25,26,27]. The review [24] also highlighted a positive impact on overall quality of life and a reduction in sleep disturbances.

Despite its promise, research on the effectiveness of online yoga in cancer patients remains limited, highlighting a critical gap in the literature. This scarcity of research may be attributed to several factors, such as the recent rise in online yoga’s popularity, the challenges of conducting large-scale, remote studies that can ensure consistent data collection, and the prioritization of more traditional cancer therapies in research funding and focus.

This systematic review aims to address this gap by synthesizing existing evidence on the benefits of online yoga for cancer patients. Through an assessment of its effects on both physical and psychological well-being, the review aims to offer a thorough perspective on the role of online yoga as a complementary approach in oncology care while highlighting key areas for future investigation.

## 2. Materials and Methods

PRISMA Guidelines. The study followed the PRISMA 2020 guidelines for systematic reviews, ensuring thorough planning and execution. These guidelines, updated from the 2009 version, provide detailed recommendations for conducting reviews [28]. Following these guidelines, two independent authors (G.C. and B.G.) actively participated in all the stages of the systematic review, including study selection, data extraction, and quality assessment. Any disagreements at any stage were resolved through discussion between the two researchers, and if no consensus was reached, a third author (F.G.) was consulted to mediate and make the final decision.

Search Strategy. This review aims to evaluate the impact of online yoga on physical and psychological outcomes in cancer patients, providing a comprehensive understanding of its potential as a supportive intervention in oncology care and identifying priorities for future research. The research strategy and inclusion/exclusion criteria were carefully designed to align with this focus.

Potentially eligible articles were systematically searched on Medline, PsycINFO, and Scopus databases on 24 October 2024 using the following combinations of terms: “yoga” AND “online OR web-based OR remote” AND “cancer OR tumor OR oncology”. Only papers retrieved on this specific date were included in the search process. We limited the search by incorporating a filter into the databases, restricting it to publications written in English due to the language proficiency of our research team. Subsequently, the articles were manually selected. Finally, because clinical interest in the topic addressed by the review has emerged recently, no filters were used for publication date.

Inclusion and Exclusion Criteria. We set the following inclusion criteria:

- Studies had to report on primary research;
- Studies had to be published in a scientific journal (e.g., no dissertations or books);
- Studies had to be written in English;
- Studies had to include only adult subjects (18+);
- Studies had to include only subjects with a cancer diagnosis;
- Studies had to include patients engaged in online yoga sessions.

We set the following exclusion criteria.

According to the methodology of the study, the following papers were excluded:

- Studies that proposed theories, models, guidelines, or protocols but did not assess the efficacy of the intervention;
- Studies on the intervention’s usability;
- Reviews and meta-analyses;
- Preliminary studies and single-case studies.
- Regarding the practice of yoga, the following papers were excluded:
- Studies that do not separately assess and distinguish yoga outcomes from other practices.
- Studies where yoga was conducted both in person and online.

Methodological Quality Assessment. Oncological research, especially from a psychological perspective, faces unique challenges due to the subjective nature of evaluating nonpharmacological therapies, which are harder to measure precisely compared to pharmacological interventions. The limited and less robust literature reflects the newness of nonpharmacological approaches in supportive oncology care. As a result, studies were not required to meet a predefined quality threshold for inclusion.

Nevertheless, assessing their methodological quality was crucial to identifying potential limitations and appropriately weighing the results. The evaluation was conducted by two independent reviewers (BG and GC) using the Mixed Methods Appraisal Tool (MMAT) corroborated by the Critical Appraisal Checklists (CASP) for quality assessment [29,30]. Quality was designed as high (>90% criteria met), moderate (50–90%), or low (<50%). The assessment of methodological quality is provided in the table below (Table 1 and Table 2). Further details on the assessment can be found in the Supplementary Materials Table S1.

Categorization of the Study. Based on the selected study, the most frequently measured outcomes were pain, anxiety, depression, fatigue, stress, and sleep. Additionally, there were secondary outcomes that were not fully explored, as they were reported in only a small number of studies.

Conducting an in-depth analysis of these secondary outcomes was deemed unnecessary for several reasons. First, the limited number of studies reporting these outcomes made it challenging to draw meaningful or generalizable conclusions. Analyzing outcomes with such sparse data risks producing results that are highly context-dependent and not representative of broader trends. Second, reporting these individual results would have introduced the potential for misinterpretation, as isolated findings may appear disproportionately significant without the support of robust data. Finally, attempting to integrate such limited data could have undermined the overall integrity of the review, as it might dilute the analysis with unreliable or fragmented findings. Reporting individual results was not only unfeasible but could also have been misleading and risked compromising the integrity of the review. For these reasons, the review concentrated on the primary outcomes, ensuring a more coherent and reliable synthesis of the evidence. Moreover, primary outcomes were also evaluated because of their direct relevance to the quality of life and overall well-being of cancer patients undergoing yoga interventions.

Data were systematically extracted and arranged in a table format. For this systematic review, two independent researchers (B.G. and G.C.) gathered the data, each performing the extraction separately to reduce potential bias. Any disagreements between the reviewers were addressed through discussion, and consensus was reached on all data points.

## 3. Results

**Figure 1:**
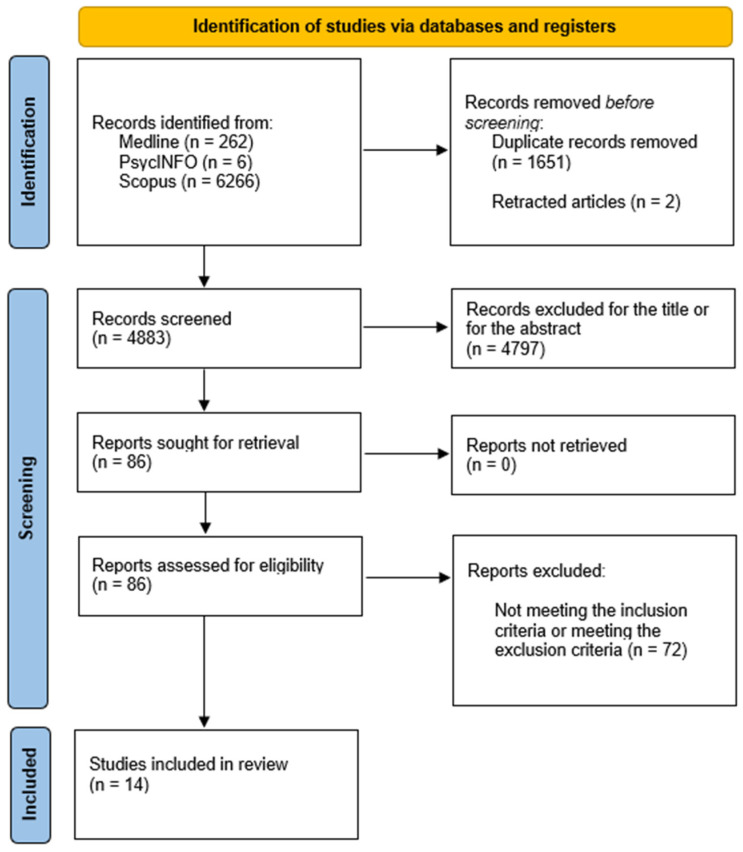
PRISMA flowchart summarizing study selection process.

Studies Selection. A total of 6498 articles were identified through the search, with two retracted by authors and 1651 duplicates removed using Zotero software version 6.0.19 and version 7.0.3. In the first stage, two independent researchers (BG and GC) screened the titles and abstracts of the papers found. Specifically, the two researchers screened 4883 articles. Simply by reading the titles, 4797 papers were removed. After screening the abstract, another 36 articles were removed. Full-text screening of the remaining 86 studies resulted in the inclusion of 14 studies for the review (Figure 1). When discrepancies were identified between the two researchers, they discussed the article to reach a consensus.

Characteristics of the Studies. The included studies were conducted globally, though four of them featured heterogeneous samples [31,32,33,34], while the remaining ten had homogeneous cohorts. Among those that specify, there are studies on patients with breast cancer (n = 9), malignant head and neck tumors (n = 3), lung cancer (n = 3), colorectal cancer (n = 3), gynecological cancer (n = 3), myeloproliferative neoplasms (n = 3), central nervous system cancer (n = 3), hemato-oncological malignancies (n = 3), skin cancer (n = 3), pancreatic cancer (n = 2), gallbladder cancer (n = 2), thyroid cancer (n = 2), colorectal cancer (n = 1), genitourinary cancer (n = 1), and allogenic bone marrow cancer (n = 1). Most studies included in this review are randomized control trials (n = 6), the others are pre-post studies (n = 8).

Among the 14 studies, demographic and clinical details were variably reported. Twelve studies provided age information, with the mean age of participants ranging from 34 to 60 years, while two studies did not specify age. Across all studies, participants were adults aged 18 years or older. Gender distribution was detailed in most studies, with several focused exclusively on female populations due to the nature of the cancer studied (e.g., breast cancer). Educational attainment was reported in most studies, with a substantial proportion of participants holding college degrees. Overall, educational levels varied, but studies frequently reported high proportions of participants with advanced education.

Other variables, including marital status, ethnicity, BMI, treatment conditions, and time since diagnosis, were reported inconsistently and were not available for all studies. For example, some studies specified whether interventions were conducted on or off medication, while others omitted this information. Adverse effects and dropout rates were occasionally discussed but not systematically across all studies. This variability in reporting underscores the need for cautious interpretation of the findings and highlights areas where future research could improve data consistency.

The studies included in this review demonstrated significant variability in both the intensity and frequency of yoga sessions. Most interventions were delivered over a period ranging from 4 to 12 weeks, with weekly sessions being the most commonly used format. The structure of these sessions varied widely, with some programs offering structured group classes, typically lasting 1.5 h, while others implemented 60 min yoga practices followed by relaxation [42,44]. In contrast, other interventions were home-based or video-guided, with daily short videos (e.g., 20 min practices) delivered over 4 weeks, or weekly 60 min online sessions, where participants followed the instructions independently.

Some studies used hybrid models, combining in-person group sessions with home-based or video-guided practices. For example, one eight-week program blended weekly somatic yoga and meditation sessions with daily home practice, providing a comprehensive approach that allowed for both group interaction and independent practice [36,44]. On the other hand, some studies relied solely on digital formats, such as pre-recorded video series or interactive online sessions.

The duration of each session varied widely across studies, ranging from as short as 10 min [32] to up to 2 h [40], depending on the design of the intervention and the specific needs of the target population. These variations in session duration, frequency, and format highlight the adaptability of yoga interventions to different contexts, populations, and logistical constraints.

While most studies included in this review provided clear descriptions of the session protocols, some lacked consistency in reporting specific details on session frequency and intensity. This inconsistency may reflect the diverse nature of the studies included and the flexibility with which yoga interventions are implemented. Further details regarding the session protocols, including their frequency and intensity, are summarized in Table 3 and discussed in greater detail in the Results section.

Our review encompassed both quantitative and qualitative research, comprising 10 quantitative studies and 4 qualitative studies to provide a comprehensive and in-depth understanding of the effectiveness of online yoga, combining numerical data with participants’ subjective experiences. This approach allowed us to corroborate quantitative findings with individual perceptions, offering a more nuanced assessment of the impact of online yoga on cancer patients’ health.

Additional details are available in Table 3 for quantitative studies and Table 4 for qualitative ones.

### 3.1. Pain

Five studies investigated the effectiveness of online yoga interventions for pain management in cancer patients [34,35,36,37,38,39,40]. The results show variability, with no consistent evidence of significant pain reduction.

One study focused on patients who underwent bone marrow transplant, reported a modest 6.1% improvement in pain scores from baseline to follow-up, but the effect size was very small (η^2^ = 0.007), suggesting no significant clinical relevance (*p* = 0.53) [35]. Another study found a small increase in back pain severity (*p* = 0.041), though this may have been influenced by factors such as musculoskeletal comorbidities or delayed muscle soreness [36]. Indeed, a qualitative study found that only a minority of myeloproliferative neoplasms (MPN) participants reported positive effects, such as pain reduction, while the majority did not experience significant relief, and some found the yoga sessions physically uncomfortable [38].

Moreover, no significant changes were found in clinician-measured or patient-reported pain outcomes, including Brief Pain Inventory (BPI) severity and interference scores (*p* > 0.609). Similarly, in another study, the assessment of pain severity and interference using the BPI revealed no significant changes after the yoga program in breast cancer patients, with small effect sizes and non-significant *p*-values (*p* > 0.05) [40]. In terms of pain intensity, online yoga interventions produced small reductions in pain scores over time, but these changes were not statistically significant [37,39]. Finally, no significant changes in pain were found across three measurements from baseline to week 8 and week 16, following the online yoga intervention conducted via videoconference (*p* = 0.770) [34].

### 3.2. Anxiety

In addition to pain, anxiety is one of the most studied variables in the context of online yoga interventions for cancer patients [32,33,35,37,39,40,41]. Of the seven studies reviewed, three reported significant reductions in anxiety levels following online yoga interventions, suggesting that online yoga could potentially offer therapeutic value for anxiety reduction in cancer patients [37,40,41].

One study showed a statistically significant reduction in generalized anxiety disorder (GAD) scores at all time points, with an average reduction of 2.9 points by the end of the study. The most notable improvement was observed between week 0 and week 4, indicating a gradual effect over time, following the mindful-based intervention with internet-streamed yoga videos in breast cancer survivors (t = −2.97, *p* = 0.004) [41]. A second study also found significant reductions in anxiety at weeks 12 (d = −0.67, *p* = 0.002) and 16 (d = −0.54, *p* = 0.02), with continued improvement over time, after a 12-week home-based online yoga intervention. [37]. Additionally, another study, focused on breast cancer patients, reported significant reductions in state anxiety through a mobile mindfulness-based stress reduction program, with a notable effect size of 0.72 (*p* = 0.03) [40].

However, not all studies found such consistent effects. Four studies reported no significant improvements in anxiety levels following online yoga interventions, particularly when compared to other forms of intervention or standard care [32,33,35,39]. One study, conducted on patients post-bone marrow transplant, found that while both the yoga and control groups showed a reduction in anxiety, the effect was slightly stronger in the control group, raising questions about potential external factors influencing the outcome [35]. Similarly, another study observed small effect sizes for anxiety reduction in the yoga group compared to a control group that maintained their usual activity for 16 weeks, though these were not statistically significant [39]. Two additional studies found no significant changes in anxiety levels, with one showing no difference between the intervention and control groups (GAD-7, *p* = 0.75) [32] and another reporting no significant change in anxiety symptoms at three months post-intervention (d = −0.09, *p* = 0.463) [33].

### 3.3. Depression

This systematic review analyzed six studies investigating the effects of online yoga on depression in cancer patients, indicating mixed results [32,33,35,37,39,40]. Three studies reported significant reductions in depression following online yoga sessions, suggesting a beneficial effect for some patients [33,37,40]. In contrast, the other three studies found no significant improvement in depression, suggesting that online yoga may not offer substantial benefits for managing depressive symptoms in all cancer patients. [32,35,39].

One study, conducted on participants with breast cancer, demonstrated significant psychological improvements in participants, with depression scores notably decreasing after six weeks of online yoga. The results suggest a substantial positive impact on depression reduction (*p* = 0.01) [40]. Another study reported a significant reduction in depression three months after the video-based intervention (d = −0.27). However, although there was a slight but significant reduction in depressive symptoms between baseline and follow-up, no significant changes were observed between post-intervention and follow-up (*p* = 0.463) [33]. Another study found a significant reduction in depression after 12 weeks of online yoga, (d = −0.41, *p* = 0.049), and further improvements at week 16 (d = −0.62, *p* = 0.004) [37].

On the other hand, in one study conducted on patients post-bone marrow transplant, depression scores decreased in both the yoga and control groups, but the overall effect was minimal, with a slight reduction in scores observed in both the control and yoga groups. (η^2^ = 0.001, *p* = 0.4339) [35]. A similar trend was observed in a follow-up study by the same research team, where the online yoga intervention led to reductions in depression scores among participants. Over 16 weeks, the Yoga Group showed consistent improvement (d = −0.64 to −0.78), indicating medium to large effects. In contrast, the Control Group experienced small increases or minimal changes in depression scores across all time points [39]. Lastly, one study found no significant differences between the intervention and control groups, with depression scores decreasing in both groups but no significant difference in the mean change (*p* = 0.954) [32].

### 3.4. Fatigue

This systematic review analyzed eight studies investigating the impact of online yoga on fatigue among cancer patients, resulting in mixed findings [32,33,34,35,37,38,39,40].

Several studies reported significant improvements in fatigue levels following online yoga interventions, supporting its therapeutic potential. For instance, one study demonstrated a significant reduction in fatigue after 12 weeks of yoga, with an average decrease of 0.8 points from baseline (*p* = 0.04) [37]. Similarly, another study reported significant improvements in both psychological and physical symptoms of fatigue among breast cancer patients after six weeks of yoga, with a notable reduction in fatigue scores (*p* = 0.002) and a decrease in fatigue’s interference with quality of life (*p* = 0.03) [40]. This was further supported by qualitative data from a study that found MPN participants subjectively reported improvements in overall physical condition, with nearly half of them mentioning a reduction in fatigue symptoms [38]. Additionally, a reduction in fatigue was observed in the yoga group, although the difference was not statistically significant when compared to the control group. Nonetheless, the effect size was modest (d = −0.56), suggesting a potential benefit [39]. Furthermore, a significant reduction in fatigue was found three months after completing a video-based yoga intervention, though the effect size was small (d = −0.24, *p* = 0.01) [33]. Another study showed moderate improvements in fatigue post-intervention, but these changes were not statistically significant (*p* = 0.097) [34].

### 3.5. Stress

This systematic review examined five studies on the effects of online yoga on stress among cancer patients [34,36,38,40,42]. Overall, most of the evidence supports the efficacy of online yoga in managing stress among cancer patients.

In one study, the impact of an 8-week somatic yoga and meditation program on stress levels was assessed. Although a reduction in stress was observed, the change was not statistically significant (*p* = 0.608) [36]. Another study, focused on breast cancer patients, showed significant improvements in perceived stress. Perceived Stress Scale scores decreased from baseline after six weeks (*p* = 0.004), with a large effect size (d = 0.84) [40]. One more study evaluated the impact of an online yoga intervention on perceived stress, finding that stress levels significantly decreased from baseline to post-intervention and remained lower at follow-up (*p* = 0.011), with a moderate effect size (ηp^2^ = 0.170) [34].

Finally, two qualitative studies have supported these hypotheses. In one study, most participants reported mental health benefits, particularly a reduction in stress, with 14 individuals specifically noting an improved ability to manage stress through yoga. Additionally, two MPN participants initially experienced stress at the beginning of the program, which decreased over time [38]. The other study, which examined the effects of a three-month vinyasa yoga program during COVID-19-induced self-isolation, on breast cancer patients, found that the participants’ expectations for reducing stress were fully met, suggesting that vinyasa yoga was effective in managing stress and tension associated with cancer and its treatment [42].

### 3.6. Sleep

Seven studies investigated the effectiveness of online yoga interventions for sleep in cancer patients [36,37,38,39,40,42,43], with only one study reporting no significant results, though its findings were still generally positive [36], suggesting that yoga may have a positive effect on sleep quality.

One study demonstrated a significant improvement in overall sleep quality after 6 weeks of yoga in breast cancer patients, (*p* = 0.007) compared to the control group, with notable improvements in sleep disturbance, sleep latency, and daytime sleepiness. Additionally, 19.7% of participants shifted from clinically significant sleep disturbance to normal sleep scores, and 12% of participants with severe sleep disturbance showed improvement, compared to only 4.8% in the control group [43]. Another three studies consistently demonstrated the positive impact of online yoga on sleep [37,38,39]. One study found significant improvements in sleep disturbance over 12 weeks (*p* < 0.001) [37]. Another study further supported these findings, showing moderate reductions in sleep disturbance over 16 weeks, with effect sizes ranging from −0.26 to −0.61 across different time points, and significant improvements in the yoga group compared to the control group [39]. One of the studies also echoed these results, with MPN participants reporting improved sleep as one of the most common physical health benefits [38].

A pilot study on a mobile mindfulness-based stress reduction program that included yoga components revealed significant improvements in sleep quality, particularly in reducing daytime dysfunction, with a medium effect size (d = 0.65, *p* = 0.01) [40]. Another study, examining the effects of a three-month vinyasa yoga program on breast cancer patients during the COVID-19 pandemic, found notable improvements in sleep difficulties post-intervention, with participants reporting a decrease in sleep problems and a significant portion (61.54%) indicating that they slept better after completing the yoga course [42]. Finally, one study provided weekly somatic yoga and meditation sessions over eight weeks, with an additional home program component. Although slight improvements in sleep quality were observed, the change was not statistically significant (*p* = 0.644) [36].

### 3.7. Other Outcomes

This systematic review primarily focused on pain, anxiety, depression, fatigue, stress, and sleep, as these outcomes are closely linked to the quality of life in cancer patients undergoing online yoga interventions. These factors were consistently addressed across the studies reviewed. In contrast, other outcomes such as psychological well-being, mindfulness, and physical function were examined in few studies [34,38,41,43,44]. Because these additional outcomes were evaluated in isolated studies, drawing definitive conclusions from these findings may not be appropriate.

Other potentially relevant factors, such as flexibility, balance, gait speed, and muscle strength for physical function, or fear of falling, spiritual well-being, and sexual satisfaction for psychological well-being, were not the primary focus of the studies reviewed. Overall, the findings suggest that online yoga interventions primarily target pain and psychological distress, which are central to the well-being of cancer patients. However, other factors could also contribute to the overall effect of yoga on patients’ quality of life, and further research is needed to better understand their significance.

## 4. Discussion

Cancer significantly impacts patients’ quality of life, often causing psychological, emotional, and social issues such as anxiety, depression, stress, fatigue, and sleep disturbances. These challenges are frequently underestimated compared to physical symptoms [45]. Complementary therapies like yoga are gaining recognition for their role in addressing these issues through physical activity, breathing exercises, and meditation [46].

However, traditional in-person yoga poses accessibility barriers for many cancer patients, including those with mobility limitations caused by the cancer itself or its treatments, demanding schedules due to frequent medical appointments, and geographical constraints. Online yoga offers a practical and inclusive alternative, enabling patients to participate from home while addressing these barriers [47].

This systematic review specifically aimed to evaluate the effects of online yoga practice on cancer patients, focusing on outcomes such as pain, anxiety, depression, fatigue, stress, and sleep. Despite the inherent limitations of remote practice, online yoga holds promise as a flexible, cost-effective, and widely accessible intervention, particularly for patients who may not have access to traditional in-person sessions. Our findings suggest that online yoga may be a beneficial intervention for addressing these common challenges, particularly in improving psychological well-being and managing symptoms like stress and sleep disturbances, although its effectiveness varies across studies. Moreover, the integration of online yoga into oncology care aligns with the growing fields of integrative medicine and telehealth [23], underscoring its potential as a low-cost, non-invasive approach to support physical and mental health. Existing evidence supports the feasibility and acceptability of online yoga interventions [23], suggesting that they could become a viable component of routine clinical care. However, this review also highlights the need for further exploration of delivery methods and intervention characteristics to optimize effectiveness. The current literature shows a significant gap in understanding how delivery methods influence outcomes, with limited attention paid to population-specific needs and detailed reporting of intervention characteristics [48]. Furthermore, the challenge in fully exploring the effectiveness of online yoga is evident, as much of the existing research remains superficial. Non-pharmacological treatments, such as yoga, are still a relatively recent proposal in the field of oncology supportive care [49]. This suggests that the current body of evidence is still emerging and not yet fully comprehensive. The limited number of studies and their methodological constraints contribute to this superficial exploration, emphasizing the need for more rigorous and expansive research to better understand the effectiveness of online yoga as part of cancer care. Future research should address these gaps, accounting for barriers specific to diverse populations and prioritizing the development of guidelines that enhance intervention efficacy.

One potential limitation of online yoga classes is the reduced level of interaction among participants. Several studies included in this review [31,32,33,34,38] note that interaction may be an important variable, with the potential to influence the effectiveness of these interventions and enhance their benefits. Human connection plays a vital role by fostering emotional support, a sense of belonging, and shared motivation, all of which are key to improving mental and physical outcomes. Interestingly, two studies [31,42] conducted during the COVID-19 pandemic offered valuable insights into this issue. While they did not observe specific effects, they noted that online yoga sessions provided much-needed human contact during a time when physical distancing measures severely limited social interaction. In conclusion, future studies should aim to more precisely evaluate the role and impact of human interaction in online interventions. Gaining a deeper understanding of how human connection influences engagement, motivation, and overall effectiveness could help develop more impactful and supportive online programs, addressing both the therapeutic and social needs of participants.

It is well-known that regular yoga improves posture, body alignment, muscle strength and flexibility, which may reduce muscle tension and pain caused by stiffness. Additionally, yoga boosts the release of endorphins, providing natural pain relief like other forms of exercise [50]. However, its effectiveness can diminish, and the risk of injury can increase when postures are practiced at home without proper supervision. Studies on cancer-related pain exemplify this limitation, as none have reported significant improvement from online yoga practice [34,35,36,37,38,39,40]. A possible explanation could be that online formats inherently limit personalized assistance, reducing the instructor’s ability to monitor the patient’s posture and breathing. This lack of real-time feedback may impede the necessary adjustments to align the practice with the patient’s needs, potentially reducing its effectiveness in alleviating pain.

Contrasting findings suggest that online yoga may effectively manage anxiety in cancer patients, including those with myeloproliferative neoplasms [37] and breast cancer [40,41]. These studies indicate that yoga, as a general practice, can help modulate the autonomic nervous system through controlled breathing techniques, potentially lowering heart rate, and promoting relaxation. Additionally, various forms of yoga incorporate mindfulness practices that enhance present moment awareness, which may reduce anxiety associated with future worries or past events [51]. However, the effectiveness of online yoga in reducing anxiety appears to vary depending on the type of cancer and study design. For example, studies reporting significant improvements included patients with myelofibrosis, polycythemia vera, and thrombocythemia [37] or breast cancer [40,41]. Conversely, other studies either did not specify the cancer type [32,33,39] or focused on patients undergoing bone marrow transplants [35], reporting less conclusive benefits. These findings highlight the potential influence of cancer type, intervention protocols, and individual patient characteristics on the outcomes of online yoga interventions.

Online yoga’s benefits for depression present a similarly complex picture. Depression is often associated with elevated levels of cortisol (the stress hormone), and research shows that yoga can help lower cortisol levels, leading to improved mood and reduced depressive symptoms [52]. Three studies reported significant improvements in depressive symptoms due to online yoga, highlighting its potential as an accessible therapeutic tool [33,37,40]. However, other studies showed no notable changes [32,35,39], suggesting that the effectiveness of yoga for depression is not consistent across all patient groups and may be influenced by different factors. Group yoga classes offer social support and a sense of community, which can be especially valuable for those feeling isolated due to depression. In such cases, online practice may not provide the same benefits. On the other hand, despite the physical distance, online yoga can still provide some individuals with a sense of belonging and connection. Moreover, given that cancer patients often struggle with chronic fatigue, conserving energy and channeling it into wellness activities can positively impact their mood and help reduce symptoms of depression.

Regarding fatigue, findings are similarly inconsistent. Some studies highlighted significant reductions in fatigue due to online yoga [33,35,37,40], while others observed no substantial effects [32,34,38,39]. The variability in these effects among oncology patients may stem from differences in cancer type, stage, and treatment phase, all of which influence baseline fatigue levels. Additionally, patient characteristics such as physical fitness, familiarity with yoga, and psychological factors like anxiety or openness to complementary therapies may moderate the intervention’s effectiveness. Furthermore, the remote nature of online yoga may influence outcomes, as the lack of in-person supervision can affect the quality of posture alignment and engagement, potentially reducing the therapeutic benefits for fatigue management.

Stress reduction is another important area where online yoga demonstrates broad potential. As mentioned, many yoga practices incorporate mindfulness, which involves focusing on the present moment. This mindful attention can help reduce rumination and negative thoughts that often fuel stress. Online yoga has shown potential for reducing stress levels among oncology patients; however, it is important to consider the limited methodological quality of some studies included in the review [34,38,40,42].

Finally, some yoga exercises can also help regulate the circadian rhythm, the body’s internal clock affecting sleep–wake cycles. Regular practice can establish a more consistent sleep routine and improve sleep quality, inducing a state of profound relaxation that can be particularly effective in enhancing sleep quality [53]. Most studies included in this review found significant improvements in sleep quality [37,38,39,40,43], suggesting that online yoga may be useful in managing sleep problems in cancer patients, although one study reported only marginal improvements.

It is also worth discussing the findings from the four qualitative studies included in this review, as they provide valuable insights into the experiences of cancer patients participating in online yoga interventions, highlighting both the benefits and challenges of this modality. One study focused on young adults with cancer (aged 18–39) who completed an 8-week yoga intervention delivered via videoconference. The results emphasized several key themes, such as improved mental and physical health, increased self-compassion, and the importance of establishing a routine. Participants also reported feeling supported by the virtual community, suggesting that the online format can provide emotional and social benefits, in addition to the physical benefits typically associated with yoga [31]. Similarly, a study on patients with myeloproliferative neoplasms also found that an online yoga intervention can lead to improvements in physical health, such as better sleep and reduced fatigue, as well as mental health benefits, including reduced stress. Participants appreciated the convenience of practicing yoga at home, highlighting that online formats can help overcome common barriers to in-person interventions [38]. A third study, which examined the impact of an 8-week yoga program on women with breast cancer-related lymphoedema, found that yoga enhanced physical awareness, improved well-being, and fostered a sense of empowerment. The social aspect of the group, even when virtual, was also seen as beneficial, providing participants with a platform to share experiences and support one another [44]. Finally, another study focusing on breast cancer patients during the COVID-19 pandemic found that a 12-week online vinyasa yoga intervention significantly reduced stress and improved sleep quality. These benefits were particularly important during the period of self-isolation, where participants reported enhanced well-being and self-acceptance [42].

Together, these studies illustrate that online yoga interventions can be an effective and accessible complementary therapy for cancer patients, offering improvements in both physical and mental health. They highlight the potential for yoga to address common cancer-related symptoms such as fatigue, stress, and sleep disturbances, while also promoting self-care and social support. Indeed, higher perceived support predicts better physical functioning, particularly in the context of chemotherapy [54].

However, the variation in results across different cancer types and patient demographics underscores the need for further research to tailor interventions more effectively and confirm long-term benefits. In conclusion, online yoga appears to be a promising intervention for cancer patients, providing a feasible way to enhance quality of life and support overall well-being during and after treatment. Our findings suggest that online yoga has a minimal impact on pain compared to traditional yoga, with less pronounced improvements in mental health, though it consistently benefits sleep quality—an outcome also supported by studies on traditional yoga, which show mixed results overall.

Certainly, online yoga represents a viable and well-tolerated alternative for cancer patients. Online yoga interventions increase accessibility, reduce missed sessions, and are generally associated with high levels of participant satisfaction [21]. Additionally, yoga’s low cost and minimal equipment needs make it easily accessible for home practice [22]. This flexibility offers a non-invasive and adaptable approach to improving both physical and mental health, showing promise as a remote therapeutic intervention [23]. While most yoga interventions have traditionally been conducted in person, online yoga offers distinct advantages, including eliminating travel-related challenges, reducing associated costs, while maintaining high satisfaction rates among participants [21,55]. Numerous studies have shown improved quality of life scores and reduced stress levels among cancer survivors participating in yoga programs compared to waitlist controls [56]. Thus, online yoga provides a practical solution to many of the barriers that can prevent in-person participation—such as fatigue, pain, or logistical limitations—especially since not all patients diagnosed with an oncological disease have the opportunity to easily access facilities for yoga practice. Importantly, evidence from several studies highlights the safety and tolerability of online yoga interventions. Few studies specifically address the potential side effects of online yoga interventions; however, those that do have not reported any adverse events during or after the programs [31,34,36]. This absence of reported side effects in the limited available data suggests the general acceptability and safety of online yoga programs, even for populations with specific health challenges such as cancer patients. Notably, only two studies documented isolated adverse effects, reporting a case of enlarged spleen. In both instances, the yoga programs were carefully adapted to meet the individuals’ needs, successfully alleviating symptoms [37,38]. One of these studies also reported that 11 participants experienced pain or discomfort during the interventions, emphasizing the need for tailored interventions to ensure safety. These findings highlight the adaptability and general safety of online yoga, supporting its use as a safe, inclusive, and effective approach for diverse populations, including cancer patients.

In terms of participant engagement, dropout rates for online yoga interventions varied widely, ranging from 6.67% to 71.3% (as shown in Table 3 and Table 4) with the most common range occurring between 30% and 40%. These rates appear to reflect general challenges in sustaining participant engagement during interventions, rather than difficulties specific to the yoga programs or adverse effects. Factors such as personal circumstances, lack of time, or diminished interest in the intervention (e.g., perceived monotony) were commonly cited as reasons for attrition.

However, as highlighted, while online yoga shows promising benefits for certain symptoms in cancer patients, the variability in results suggests that it is not a one-size-fits-all solution. This indicates that the effectiveness of online yoga may depend on factors such as the specific type of program, the experience level of the instructor, and the individual characteristics of the participants, including their global health status, physical abilities, and personal preferences. Additionally, some patients may require more personalized or hands-on guidance to fully benefit from yoga practices.

Study limitations. Several limitations of the current literature need to be addressed, such as small sample sizes, inconsistent methodology, low quality of studies, and low statistical power. Furthermore, the heterogeneity of studies regarding intervention protocols (e.g., frequency, duration, and yoga type) and diverse outcomes challenge the synthesis of results. Moreover, the sole inclusion of English-language studies may introduce selection bias and limit the cultural and linguistic diversity of the evidence base. Future reviews should involve bilingual experts to broaden the scope of research.

Moreover, the methodological quality varied substantially between studies. Generally, qualitative studies were good in exploring patient experiences, while many cohort studies struggled with low response rates and design flaws, reducing their reliability. RCTs exhibited inconsistent quality, with few having standardized protocols and clear outcome reporting. This, therefore, indicates the urgent need for more rigorous and well-structured research to underpin the evidence base. Furthermore, the heavy reliance on self-reported measures calls for a greater emphasis on the use of objective markers, such as physiological indicators, to further capture the effects of online yoga.

Varying dropout rates also present a challenge, potentially limiting the generalizability of findings. Overall, though varied, dropout rates supported the general pattern of the long and online interventions as challenging, hence a need for future strategies to maintain participants and hold their interest. Personal issues, such as lack of time, motivation, and perceived dullness of interventions, were key determinants that influenced dropout, hence calling for interventions tailored to program needs and better engagement strategies as ways to address attrition. Furthermore, the influence of reduced human interaction in online yoga formats should be further explored, since social connection and instructor feedback are crucial in developing engagement and enhancing therapeutic benefits.

## 5. Conclusions

This review points out that online yoga has the potential to be a widely available and cost-effective intervention for cancer patients, especially for those with limited mobility, severe symptoms, or limited access to in-person care. The findings suggest promising benefits on sleep quality, reduction in stress, and fatigue, though its efficacy in pain management remains limited. Effects on anxiety and depression are variable, although online yoga appears to significantly improve quality of life, focusing on the psychosocial and emotional dimensions of care. Incorporation of qualitative studies also allows very important insights into patients’ experiences, outlining other advantages such as better emotional well-being, empowerment, and personal fulfillment. While these findings deepen our understanding of yoga’s holistic impact, further quantitative research is needed to strengthen the evidence base and ensure broader applicability.

Online yoga holds promise as a non-pharmacological intervention with minimal side effects, supporting comprehensive symptom management. Future research should strive to develop standardized protocols—including session frequency, duration, and yoga style—to ensure comparability across studies and enhance validity. Programs of personalized yoga may improve adherence and possibly therapeutic outcomes by tailoring them specifically to the needs of the patient, and considering factors like cancer type, disease stage, and comorbidities, may further improve adherence and therapeutic outcomes.

Integrating online yoga into conventional cancer care may provide a holistic approach that may address the physical, emotional, psychological, and social aspects of the disease and the patient’s experience, fostering overall well-being and recovery. More high-quality research is needed to further define benefits, limitations, and optimal implementation, ultimately solidifying online yoga’s role as a complementary therapy to improve quality of life for cancer patients and survivors.

## Figures and Tables

**Table 1:** Methodological quality evaluation.

Article	Methodology	% Yes Response	Evaluation
[31]	Qualitative	100%	High
[32]	RCT	31%	Low
[33]	Cohort	43%	Low
[34]	Cohort	43%	Low
[35]	RCT	54%	Moderate
[36]	Cohort	28.5%	Low
[37]	Cohort	78.5%	Moderate
[38]	Qualitative	80%	Moderate
[39]	RCT	77%	Moderate
[40]	Cohort	43%	Low
[41]	Cohort	78.5%	Moderate
[42]	Qualitative	80%	Moderate
[43]	RCT	69%	Moderate
[44]	Qualitative	90%	Moderate

**Table 2:** Considerations on quality assessment.

Study Type	Key Strengths	Key Limitations	Quality
Randomized Controlled Trials (RCTs)	- Clear research objectives - Randomization implemented	- Lack of blinding in several studies - Incomplete follow-up - Limited reporting of effect sizes	Moderate
Cohort Studies	- Adequate measurement of exposure and outcomes - Sufficient follow-up for most participants	- Inconsistent recruitment processes - Poor control of confounders - Limited precision in results	Low
Qualitative Studies	- Clear aims and objectives - Appropriate methodology	- Limited exploration of researcher–participant relationships	High (with minor concerns)

**Table 3:** Quantitative results of the studies.

Article	Study Type	Intervention	Structure	Sample	Measurement	Effectiveness
[43]	Randomized Controlled Trial	Home-based yoga intervention.	6 weeks of online yoga, twice a week for 45 min.	173 breast cancer patients (Drop-out: 32%).	Pittsburg Sleep Interference Scale (PSQI).	**Sleep**: significant improvement after 6 weeks of yoga (*p* = 0.007), compared to the control group which had no special assignment except for refraining from starting a new yoga practice.
[35]	Randomized Controlled Trial	Home-based intervention of hatha yoga.	Home-based program of 60 min of yoga each week for 12 h, developed by a researcher with a PhD in exercise in physiology.	72 cancer patients, after bone marrow transplant (Drop-out: Yoga group: 45.5%, Control group: 38.5%).	Lee Sympton Score (LSS); Patient-Reported Outcomes Measurement Information System (PROMIS).	**Pain:** No significant effect of group (*p* = 0.53), time (*p* = 0.1524), or group × time interaction (*p* = 0.6479). No significant differences between groups at baseline (*p* = 0.9618). **Anxiety:** No significant effect of group (*p* = 0.5607) or group × time interaction (*p* = 0.3366). Significant global effect of time (*p* = 0.0357, η^2^ = 0.12). Differences between groups at baseline (*p* = 0.0089). Small effect size (η^2^ = 0.04). The yoga group improved by 8.2% from baseline to midpoint but worsened by 4.4% from midpoint to post-intervention. No significant improvements in the control group. **Depression:** No significant effect of group (*p* = 0.4339), time × group interaction (*p* = 0.9610), or small effect size (η^2^ = 0.001). Significant main effect of time (*p* = 0.0102, η^2^ = 0.06). The control group showed a 6.6% improvement and the yoga group a 5.3% improvement from baseline to follow-up. No significant difference between groups at baseline (*p* = 0.3474). **Fatigue:** No significant effect of group (*p* = 0.3770), time (*p* = 0.0639), or group × time interaction (*p* = 0.08). Very small effects (η^2^ = 0.02). No significant improvement in either group from baseline to follow-up (*p* = 0.0707).
[41]	Cohort Study	Mindful-based intervention with Internet-streamed yoga video.	Telephone interviews after 2 and 4 weeks of practicing a 20 min yoga video, daily for 4 weeks.	43 breast cancer survivors (Drop-out: 16.67%).	Generalized Anxiety Distress Scale (GAD).	**Anxiety**: statistically significant reduction at all time points. Average reduction: 2.9 points by the end of the study. Notable improvement between week 0 and week 4 (t = −2.97, *p* = 0.004).
[36]	Cohort Study	Somatic yoga and meditation intervention, incorporating neurophysiology, psychophysiology and mindfulness for increased mind–body integration.	Somatic yoga and meditation provided weekly for 8 weeks, with an additional home program component.	8 cancer patients: breast cancer (n = 4) colon cancer (n = 2) ovarian cancer (n = 1) pancreatic cancer (n = 1) (Drop-out: 71.3%).	Brief Pain Inventory (BPI); Perceived Stress Scale (PSS); Pittsburg Sleep Interference Scale (PSIQ).	**Pain**: Slight increase from 3.50 to 3.75 (*p* = 0.041). **Stress**: No significant change (from 15.75 to 15.00, *p* = 0.608). **Sleep**: No significant improvement (from 9.75 to 9.38, *p* = 0.644).
[37]	Cohort Study	Online Yoga intervention among MPN patients.	A 12-week, home-based, online yoga intervention among MPN patients, to examine the feasibility (i.e., acceptability, demand, practicality), to explore the preliminary effects of yoga on symptoms.	38 cancer patients: Polycythemia Vera (n = 16) Essential Thrombocythemia (n = 16) Myelofibrosis (n = 6) (Drop-out: 30.91%)	National Institutes of Health Patient-Reported Outcomes Measurement Information System (NIH PROMIS); The Myeloproliferative Neoplasm Symptom Assessment Form Total Symptom Score (MPN-SAF TSS).	**Pain**: No significant improvements from baseline to week 12 (d = −0.01), (*p* = 0.94) and week 16 (d = −0.18), (*p* = 0.34) **Anxiety**: Significant reduction at both week 12 (d = −0.67), (*p* = 0.002) and week 16 (d = −0.54), (*p* = 0.02). **Depression**: Significant reduction after 12 weeks of online yoga, with the mean score decreasing from 47.7 to −2.9 (d = −0.41), (*p* = 0.049), week 16 (d = −0.62), (*p* = 0.004) **Fatigue**: Significant reduction after 12 wees, with an average decrease of 0.8 points (d = −0.33), (*p* = 0.04), week 16, with an average reduction of 0.9 points, (d = −0.34), (*p* = 0.06). **Sleep**: Significant improvements over 12 weeks, with mean scores from 49.7 to 45.9. Moderate effect size (d = −0.61), (*p* < 0.001). **Adverse event**: enlarged spleen: n = 1
[39]	Randomized Controlled Trial	Online Yoga intervention among MPN patients.	Participants randomized to the online yoga group were asked to complete 60 min/week of home-based, online-streamed yoga for 12 weeks. Participants were asked to complete 60 min of yoga each week.	48 cancer patients (Drop-out: 22.58%).	Multifactor MPN Symptom Assessment Form (MPN-SAF); National Institutes of Health Patient Reported Outcomes Measurement Information System (NIH PROMIS).	**Pain**: Baseline: Yoga Group (n = 27) had a mean score of 45.1 (SD = 8.6), Control Group (n = 21) had 40.4 (SD = 9.0). Week 7: Yoga Group reduced by −1.6 (SD = 5.8), Control Group increased by 0.6 (SD = 7.5) (d = −0.34). Week 12: Yoga Group reduced by −2.4 (SD = 7.0), Control Group increased by 0.6 (SD = 6.6) (d = −0.43). Week 16: Yoga Group reduced by −3.2 (SD = 7.3), Control Group increased by 0.8 (SD = 8.4) (d = −0.51). **Anxiety**: Baseline: Yoga Group (n = 27) had a mean score of 54.0 (SD = 7.8), Control Group (n = 21) had 50.2 (SD = 8.6). Week 7: Yoga Group reduced by −2.1 (SD = 5.0), Control Group decreased by −0.7 (SD = 5.4) (d = −0.27). Week 12: Yoga Group reduced by −3.1 (SD = 5.7), Control Group decreased by −1.3 (SD = 7.1) (d = −0.30). Week 16: Yoga Group reduced by −4.2 (SD = 5.0), Control Group decreased by -i2.0 (SD = 7.2) (d = −0.37). **Depression**: Baseline: Yoga Group (n = 27) had a mean score of 49.3 (SD = 7.9), Control Group (n = 21) had 45.2 (SD = 6.0). Week 7: Yoga Group reduced by −3.7 (SD = 6.3), Control Group increased by 0.3 (SD = 5.4) (d = −0.64). Week 12: Yoga Group reduced by −3.8 (SD = 5.4), Control Group increased by 1.6 (SD = 7.7) (d = −0.78). Week 16: Yoga Group reduced by −4.4 (SD = 7.0), Control Group reduced by −0.6 (SD = 7.1) (d = −0.53). **Fatigue**: Baseline (0–10): Yoga Group (n = 27) had a mean score of 5.4 (SD = 2.3), Control Group (n = 21) had 4.5 (SD = 2.8). Week 7: Yoga Group reduced by −0.7 (SD = 2.5), Control Group reduced by −0.8 (SD = 3.1) (d = 0.02). Week 12: Yoga Group reduced by −0.5 (SD = 2.2), Control Group reduced by −0.6 (SD = 2.3) (d = 0.06). Week 16: Yoga Group reduced by −0.7 (SD = 2.4), Control Group increased by 0.6 (SD = 2.4) (d = −0.56). **Sleep**: Baseline: Yoga Group (n = 27) had a mean score of 50.1 (SD = 6.2), Control Group (n = 21) had 48.8 (SD = 6.7). Week 7: Yoga Group reduced by −2.3 (SD = 5.8), Control Group increased by 0.2 (SD = 5.2) (d = −0.44). Week 12: Yoga Group reduced by −2.5 (SD = 5.9), Control Group increased by 1.0 (SD = 4.7) (d = −0.61). Week 16: Yoga Group reduced by −3.8 (SD = 7.6), Control Group reduced by −2.1 (SD = 5.0) (d = 0.09).
[40]	Cohort Study	A standardized, stress reducing intervention that combines sitting and walking meditation, body scan, and yoga.	The mMBSR(BC) program was designed to deliver six weekly 2 h sessions via the iPad in formal meditative techniques via audio and video files, which were led through one-way interaction by a clinical psychologist trained in MBSR.	15 breast cancer patients (Drop-out: 13.33%).	Brief Pain Inventory (BPI); State scale of State Trait Anxiety Inventory (STAI-S); Center for Epidemiological Studies Depression Scale (CED); Perceived Fatigue Symptom Inventory (FSI); Stress Scale (PSS); Pittsburgh Sleep Quality Inventory (PSQI).	**Pain**: Pain Severity: Baseline: 8.93 (SD = 7.51) Week 6: 8.69 (SD = 8.17), Change: −0.24, Effect Size: d = 0.03 (*p* = 0.72), Pain Interference: Baseline: 13.73 (SD = 16.18), Week 6: 11.31 (SD = 15.07) Change: −2.42, Effect Size: d = 0.16, (*p* = 0.25). **Anxiety**: Baseline: 33.27, Week 6: 26.03, Change: −7.24, Effect Size: 0.72, (*p* = 0.03) **Depression**: Baseline: 7.47, Week 6: 4.79, Change: −2.68, Effect Size: 0.85, (*p* = 0.01) **Fatigue**: Fatigue Scores: Baseline: 15.20, Week 6: 10.77, Change: −4.43, Effect Size: 0.60, (*p* = 0.002) Fatigue Interference with Quality of Life: Baseline: 26.20, Week 6: 17.31, Change: −8.89, Effect Size: 0.47, (*p* = 0.03) **Stress**: Baseline: 14.33, Week 6: 9.08, Change: −5.25, Effect Size: d = 0.84, (*p* = 0.004) **Sleep**: Significant Improvement: Daytime dysfunction, Effect Size: d = 0.65, (*p* = 0.0). Other Sleep-Related Subdomains (Sleep duration Sleep disturbances Latency to sleep onset): No statistically significant changes, but a trend toward better sleep quality
[32]	Randomized Controlled Trial	Home-based yoga intervention.	The intervention comprised 8 videos, each about 10 to a maximum of 30 min in length. The participants were given access to a website, on which 2 video sequences were provided weekly for 4 weeks. The patients could watch them via desktop/laptop or tablet as often as they wanted.	157 cancer patients (Drop-out: 19.19%).	Generalized Anxiety Distress Scale (GAD); European Organisation for Research and Treatment of Cancer Quality of Life Questionnaire—Fatigue-12 items (EORTC QLQ-FA12); Patient Health Questionnaire (PHQ-8).	**Anxiety**: Yoga Group (n = 67), T1 mean: 6.61, T2 mean: 5.72, Mean change: 0.90 Control Group (n = 68), T1 mean: 7.00, T2 mean: 6.0, Mean change: 1.00, (*p* = 0.751) **Fatigue**: Yoga Group (n = 62), T1 mean: 36.78, T2 mean: 34.50, Mean change: 2.28 Control Group (n = 70), T1 mean: 40.60, T2 mean: 37.06, Mean change: 3.53, (*p* = 0.715) **Depression**: Yoga Group (n = 60), T1 mean: 6.97, T2 mean: 5.95, Mean change: 0.91 Control Group (n = 65), T1 mean: 7.69, T2 mean: 6.78, Mean change: 0.91, (*p* = 0.954) (T1 = before randomization; T2 = after the end of the intervention for yoga group or the waiting period for control group)
[33]	Cohort Study	Home-based yoga intervention.	Video intervention comprising eight videos that lasted for four weeks. The participants completed questionnaires at baseline (T1), after the intervention group finished, and when the control group ended their video intervention (T2). The last survey was conducted after a follow-up period three months from the end of the video intervention of each group (T3).	155 cancer patients (Drop-out: 36.36%).	Generalized Anxiety Distress Scale (GAD-7); Patient Health Questionnaire (PHQ-8); Questionnaire for parents of children with cancer, assesses physical, cognitive, and emotional aspects of cancer-related fatigue (EORTC QLQ-FA12).	**Anxiety**: (n = 96) Baseline mean: 6.57, T3:6.12, t = 1.14, (*p* = 0.259) **Depression**: (n = 98) Baseline mean: 7.09, T3: 5.76, t = 3.33, (*p* = 0.001) **Fatigue**: (n = 99) Baseline mean: 36.36, T3: 31.17, t = 2.63, (*p* = 0.01)
[34]	Cohort Study	Home-based and evidence-based therapeutic yoga program.	The yoga intervention was informed by Yoga Thrive, an evidence-based therapeutic yoga program for individuals affected by cancer and their support persons, and the expertise of the study team. Assessments were conducted at baseline (week 0), post-intervention (week 8), and follow-up (week 16).	30 cancer patients.	Functional Assesment of chronic Illness Therapy (FACIT); Multidimensional Body-Self Relations Questionnaire-Appearance Scales (MBSRQ-AS); Short Form-36 Health Status Inventory (RAND-36).	**Pain**: Baseline: Mean = 74.00, Week 8: Mean = 73.50, Week 16: Mean = 71.80, Effect size: ηp^2^ = 0.011, (*p* = 0.770) **Fatigue**: Baseline: Mean = 30.73, Week 8: Mean = 32.50, Week 16: Mean = 33.81, Effect size: ηp^2^ = 0.094, (*p* = 0.097) **Stress**: Baseline: Mean = 20.64, Week 8: Mean = 19.36, Week 16: Mean = 17.80, Effect size: ηp^2^ = 0.0170, (*p* = 0.011)

**Table 4:** Qualitative results of the studies.

Article	Study Type	Intervention	Structure	Sample	Measurement	Effectiveness
[38]	Qualitative Study	Online yoga intervention among MPN patients.	12-week feasibility study for online yoga.	39 MPN patients.	Qualitative interviews (narrative, subjective data).	**Pain**: Negative physical impacts (e.g., pain, discomfort, tiredness, over-demand): n = 11 Positive physical benefits (e.g., reduced pain): n = 3 **Fatigue**: half participants indicated that their fatigue symptoms had been somewhat alleviated by practicing yoga **Stress**: participants reported reduction in stress: n = 14, Improved ability to manage stress: n = 14, Initial stress experienced: n = 2 **Sleep**: participants reported improved sleep as one of the most common physical health benefits from the yoga program. **Adverse event**: enlarged spleen: n = 1
[44]	Qualitative Study	Home-based yoga intervention	An 8-week yoga intervention based on Satyananda Yoga, which included breathing exercises, gentle repetitions of adapted physical movements with individual modifications, followed by periods of rest, meditation, and relaxation practices. The participants attended a weekly 1.5 h class and used a 45 min DVD for daily home practice. The DVD was a modified version of the full class, featuring a reduced number of postures, a shortened introductory breathing session, and a brief final relaxation.	28 breast cancer patients (No participants lost to follow-up).	Qualitative interviews (narrative, subjective data).	Well-being; Awareness of body and physical functioning; Mental health and social functioning.
[31]	Qualitative Study	Home-based yoga intervention.	8 weeks yoga intervention delivered by videoconference.	28 cancer patients: breast cancer (n = 12); blood cancer (n = 8); thyroid cancer (n = 2); (Drop-out: 6.67%).	Qualitative interviews (narrative, subjective data).	Self-care; Self-compassion; Mindfulness; Feelings of physical competence; Establishing a routine; Being around similar others.
[42]	Qualitative Study	Online yoga session.	Three month of online Vinyasa yoga sessions, every Wednesday at 4 p.m. carried out online via the Zoom application. The 60 min vinyasa practice was followed by 15 min relaxation (Shavasana).	41 breast cancer patients (Drop-out: 68.29%).	Ad hoc pre- and post-intervention (1 = never and 4 = very often) surveys to evaluate the impact of vinyasa yoga practice on breast-cancer patients. It included three sections: health-related issues and yoga experience, quality of life, and sociodemographic questions.	**Stress:** Participants reported that vinyasa yoga effectively reduced stress and tension related to cancer and its treatment, as confirmed by pre- and post-intervention questionnaires. **Sleep:** patients reported a reduction in sleep difficulties post-intervention, with 61.54% of them indicating improved sleep after completing the yoga course.

## Data Availability

No new data were created or analyzed in this study. Data sharing is not applicable to this article.

## Supplementary Materials

The following supporting information can be downloaded at: https://www.mdpi.com/article/10.3390/healthcare13030225/s1, Table S1: CASP evaluation.
